# Age is the wrong axis: rethinking surgical selection for octogenarians with early-stage non-small cell lung cancer

**DOI:** 10.3389/fsurg.2026.1961761

**Published:** 2026-10-01

**Authors:** Vasiliki Androutsopoulou, Prokopis-Andreas Zotos, Dimitrios E. Magouliotis

**Affiliations:** 1 Department of Cardiothoracic Surgery, Faculty of Medicine, University of Thessaly, Biopolis, Larissa, Greece; 2 Department of Cardiac Surgery Research, Lankenau Institute for Medical Research, Wynnewood, PA, United States

**Keywords:** early-stage lung cancer, frailty, geriatric assessment, non-small cell lung cancer, octogenarians, patient selection, shared decision-making, sublobar resection

## Abstract

Lung cancer is increasingly a disease of older age, and as low-dose computed tomography screening extends into the ninth decade, a growing number of octogenarians present with early-stage, potentially curable non-small cell lung cancer. Faced with these patients, clinicians repeatedly ask a single question: is this patient too old for surgery? Recent prospective data show that selected octogenarians achieve long-term survival comparable to younger patients despite substantially higher complication rates, with quality of life that recovers over the first postoperative year. Yet the favorable result is attributed to careful selection that is rarely defined, and is seldom accompanied by any account of the patients who were assessed and not offered an operation. This article argues that chronological age is the wrong axis for the decision. Physiologic reserve, measurable through validated frailty instruments and established physiologic testing, predicts postoperative outcome far better than birth year, and aging is sufficiently heterogeneous that any age threshold misclassifies patients in both directions. A substantial competing risk of non-cancer death, compounded by overdiagnosis among screen-detected tumors, tempers the survival gain expected from aggressive treatment and makes overtreatment as real a hazard as age-based exclusion. A graded menu of options now exists, extending from minimally invasive lobectomy through sublobar resection and stereotactic ablative radiotherapy to active surveillance, and intensity should descend with reserve rather than with age. The task is to operationalize careful selection through structured geriatric assessment, multidisciplinary collaboration, prehabilitation, and shared decision-making, and to judge success by outcomes that older patients actually value.

## Introduction

Lung cancer remains the leading cause of cancer death, and both its incidence and the population at risk rise sharply with age (1). It is, in essence, a disease of older adults (2). The reach of early detection has widened in parallel: current guidance recommends annual low-dose computed tomography screening up to the age of 80 (3), so a growing number of patients in their ninth decade are now identified with small, early-stage, potentially curable tumors. Demographic aging will amplify this trend, and the octogenarian with a screen-detected nodule is no longer an exception in the thoracic clinic but an increasingly ordinary referral. The clinician who meets these patients tends to ask one question above all others, namely whether the patient is simply too old to operate.

Recent prospective evidence reframes that question. In a cohort of octogenarians undergoing resection for early-stage non-small cell lung cancer, postoperative complication rates were considerably higher than in younger patients, yet long-term survival was comparable and quality of life recovered over the following year, with the authors attributing the favorable outcome to careful patient selection (4). That finding is encouraging and clinically important, but it also carries an interpretive risk. A result obtained in a selected population cannot be generalized to the unselected population unless the selection itself is described. The phrase therefore does substantial work, yet it is seldom defined or operationalized, and it is rarely accompanied by an account of the patients who were considered and not offered an operation.

The difficulty is compounded by the fact that aging is not uniform. Two patients of identical chronological age may differ profoundly in muscle mass, nutritional status, cognition, and social support, and it is this heterogeneity, rather than the birth year they share, that determines how each will tolerate a thoracotomy or a period of single-lung ventilation. Any decision rule that treats an age band as a homogeneous category will therefore misclassify patients in both directions, denying an operation to the fit and offering one to the exhausted (5, 6).

In this article we argue that chronological age is the wrong axis for the surgical decision. The more useful framing is to match the intensity of treatment to a patient's physiologic reserve and personal priorities, and to convert careful selection from an adjective into a reproducible process. We first examine why age is a poor proxy for surgical risk, then consider the competing-risk problem that shapes the benefit of treatment in this age group, outline the graded therapeutic options now available, and propose how selection might be operationalized and audited.

## Chronological age is a poor proxy for risk

Age correlates only loosely with surgical risk, because what actually predicts complications is physiologic reserve. Frailty, a measurable estimate of that reserve, independently predicts postoperative complications, prolonged length of stay, and discharge to a higher level of care, and it improves the performance of conventional preoperative risk indices (5). A robust 82-year-old may carry more reserve than a deconditioned 70-year-old, so a decision anchored to birth year discards the information that matters most.

Crucially, frailty is not an impressionistic judgment but a quantifiable construct with validated instruments. The physical phenotype defines frailty by unintentional weight loss, exhaustion, weakness, slow gait speed, and low physical activity, and independently predicts falls, disability, hospitalization, and death (6). The cumulative-deficit approach, operationalized clinically as the Clinical Frailty Scale, correlates closely with a full frailty index and predicts mortality and institutionalization better than measures of cognition, function, or comorbidity alone (7). Either family of instruments can be applied in a thoracic clinic in minutes, which removes the practical objection that reserve is harder to measure than age.

This is not merely theoretical. In thoracic populations, frailty is common and frequently unrecognized by the operating team, a phenomenon described as occult frailty. When such patients are identified through structured geriatric assessment and managed collaboratively, they can undergo resection safely, often with a greater proportion of sublobar rather than lobar operations (8). Physiologic assessment complements this picture: established guidance for evaluating fitness for radical treatment in lung cancer is built on cardiac evaluation, forced expiratory volume in one second, carbon monoxide diffusing capacity, and exercise testing, with no role for chronological age as an independent exclusion criterion (9). Reserve, not age, should therefore form the foundation of selection. Figure 1 illustrates the consequence schematically: because reserve varies widely within any age band, a threshold drawn on the age axis misclassifies patients in both directions, whereas a threshold drawn on the reserve axis separates them coherently.

**Figure 1:**
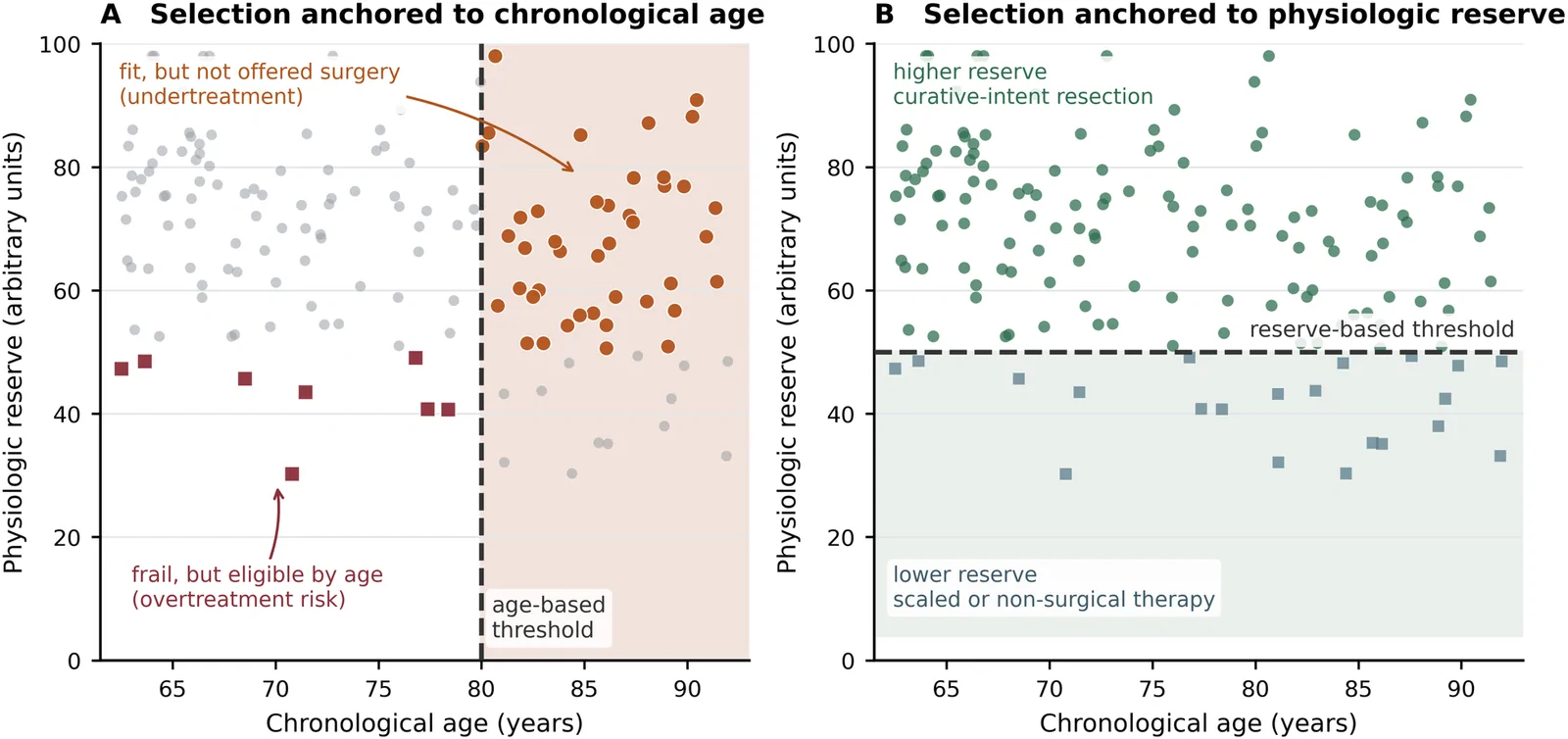
Why chronological age is the wrong axis for surgical selection. Schematic illustration of an older population with early-stage non-small cell lung cancer, in which physiologic reserve declines only weakly with age and varies widely within any age band. **(A)** A selection threshold drawn on the age axis misclassifies patients in both directions: fit patients above the age cut-off may not be offered a curative-intent operation (undertreatment, circles), while patients below the cut-off with poor reserve remain nominally eligible despite limited capacity to tolerate resection (overtreatment risk, squares). **(B)** A threshold drawn on the reserve axis separates the same population coherently, directing patients with higher reserve toward curative-intent resection and those with lower reserve toward scaled or non-surgical therapy. Data points are illustrative and do not represent an observed cohort.

## The competing-risk problem and the hazard of overtreatment

Older patients with early-stage lung cancer also face a substantial risk of dying from causes unrelated to their cancer. In a large competing-risks analysis of resected stage I disease, non-cancer mortality exceeded lung-cancer mortality for up to two and a half years after surgery in patients aged 65 and older, and this early excess grew more pronounced with advancing age (10). The practical consequence is that the survival advantage attributable to an aggressive resection is attenuated by competing mortality, and that some indolent, slowly growing screen-detected tumors may never shorten a given patient's life.

Screening itself contributes to this tension. Low-dose computed tomography reduces lung-cancer mortality but has not been shown to reduce all-cause mortality, and pooled estimates place overdiagnosis at approximately nine percent of screen-detected cancers (11). Overdiagnosis is by definition concentrated among patients whose competing risks are highest, which is precisely the octogenarian population. Overtreatment is therefore a genuine harm rather than a hypothetical one, and it is the mirror image of the undertreatment that age-based exclusion produces.

The appropriate response to either error is not therapeutic nihilism but calibration. The decision shifts from whether a tumor can be resected to whether resection will meaningfully change this particular patient's trajectory, once competing risks, tumor biology, and the patient's own goals are taken into account. Framed this way, an estimate of non-cancer life expectancy becomes as much a part of the preoperative assessment as a pulmonary function test.

Two practical implications follow. The first is that an explicit estimate of non-cancer life expectancy belongs alongside the physiologic workup, since the benefit of removing a slow-growing tumor depends on the patient surviving long enough for that removal to matter, and frailty measures predict medium-term mortality precisely in the range that is decision-relevant here (7, 10). The second is that screening and treatment decisions cannot be separated. If screening is offered into the ninth decade, the same reserve-based reasoning that governs the operation must govern the decision to look for the tumor in the first place, otherwise the pathway generates diagnoses it cannot act on proportionately (3, 11).

## A spectrum of treatment matched to reserve

Fortunately, thoracic oncology now offers a graded menu of options that can be titrated to reserve and tumor biology. For fit patients, anatomic lobectomy remains the standard, and minimally invasive approaches, whether video-assisted or robotic, reduce morbidity and protect quality of life relative to open surgery (12). Evidence specific to the oldest patients supports this: in a propensity-matched study of octogenarians with clinical stage IA disease undergoing minimally invasive sublobar resection, five-year overall survival approached ninety percent and was comparable between robotic and thoracoscopic approaches, with complication rates near twenty percent (13). Excellent outcomes in the ninth decade are therefore achievable, provided the operation is scaled appropriately.

For patients with limited reserve or small peripheral tumors, sublobar resection is no longer a compromise but an evidence-based choice: two randomized trials demonstrated that segmentectomy and sublobar resection are non-inferior to lobectomy for small peripheral tumors while preserving more lung function (14, 15). This matters disproportionately in older patients, in whom the parenchyma spared translates directly into preserved functional independence.

Scaling the operation is not the same as abandoning oncologic principle, and the distinction deserves emphasis. The randomized evidence supporting sublobar resection was generated in tumors of two centimeters or less with defined nodal staging requirements, and the equivalence it demonstrates is conditional on those constraints being respected (14, 15). Applying a lesser resection to a larger or more central tumor because a patient is elderly is not de-escalation but undertreatment. Concretely, the criteria that governed those trials should be met before a sublobar resection is considered: a peripheral tumor of 2 cm or less, a consolidation-to-tumor ratio consistent with the enrolled populations, confirmed absence of nodal disease on systematic intraoperative hilar and mediastinal sampling, and an adequate parenchymal margin, with conversion to lobectomy if these conditions are not satisfied (14, 15). Chronological age is not among these criteria, and should never be substituted for them. The octogenarian series reporting excellent long-term survival after minimally invasive sublobar resection likewise selected small clinical stage IA tumors (13), and its results should be read within those limits.

For those who cannot tolerate or decline an operation, stereotactic ablative radiotherapy provides a markedly lower-morbidity alternative with encouraging outcomes even in operable disease, and it has become a mainstay for medically inoperable patients (16). At the far end of the spectrum, for very frail patients or for clearly indolent screen-detected lesions, careful active surveillance is a legitimate option. The unifying principle is that intensity should be matched to the patient, descending from minimally invasive lobectomy through sublobar resection and radiotherapy to surveillance, rather than defaulting to the most or least aggressive option on the basis of age alone. A framework mapping physiologic reserve to treatment intensity, together with the assessment steps that assign a patient to each tier and the selection actions each requires, is proposed in Table 1. It should be emphasized that this stratification is a conceptual aid to structure multidisciplinary discussion, not a validated or prescriptive guideline. The tiers have not been derived from a prospective cohort, no threshold has been calibrated against outcome data, and the boundaries between adjacent tiers are deliberately permeable. It is offered to make the reasoning behind selection explicit and auditable, and it requires prospective validation before it could inform individual treatment decisions.

**Table 1:** A reserve- and priorities-based framework for matching treatment intensity in older patients with early-stage non-small cell lung cancer.

Reserve/frailty profile	Typical features	Suggested treatment intensity	Key selection actions
Fit, low frailty	Independent in daily activities; preserved pulmonary function; limited comorbidity	Anatomic lobectomy, minimally invasive when feasible	Standard workup; offer curative-intent surgery
Intermediate reserve or occult frailty	Borderline pulmonary function; moderate comorbidity; mild or unrecognized frailty	Sublobar resection for small peripheral tumors; minimally invasive approach	Frailty instrument; geriatric assessment; prehabilitation; multidisciplinary review
Limited reserve, frail but treatable	Reduced DLCO or FEV1; significant comorbidity; measurable frailty	SABR, or sublobar resection after optimization	Formal frailty index; geriatric co-management; prehabilitation; shared decision-making
Very limited reserve or dominant competing risk	Severe frailty; limited non-cancer life expectancy; or clearly indolent screen-detected lesion	Non-surgical management or active surveillance; prioritize quality of life	Competing-risk estimate; explicit goals-of-care discussion

Shared decision-making applies across all tiers, and tier assignment is revisited after prehabilitation rather than fixed at first assessment. DLCO, diffusing capacity of the lung for carbon monoxide; FEV1, forced expiratory volume in 1 s; SABR, stereotactic ablative radiotherapy. The framework is conceptual and is intended for use within multidisciplinary review rather than as a rigid rule.

## Operationalizing careful selection

If careful selection is to mean anything, it must become a process rather than a label, with named instruments, defined thresholds, and auditable steps. Its components already exist and are evidence-based. Contemporary oncology guidance recommends that all patients older than 65 undergo geriatric assessment covering physical and cognitive function, emotional health, comorbidity, polypharmacy, nutrition, and social support, and that the findings be used to guide management rather than merely to describe risk (17). The clinical value of doing so is established: in a cluster-randomized trial in older adults with cancer, geriatric assessment with management significantly reduced serious treatment toxicity (18). In the thoracic setting specifically, structured assessment combined with multidisciplinary geriatric-thoracic collaboration identifies the occult frailty that surgical teams miss and supports safe treatment (8).

Selection should also be dynamic rather than a single verdict. Prehabilitation can enlarge the pool of eligible patients by improving functional capacity before resection (19), which reframes borderline candidacy as modifiable rather than fixed. This potential should nonetheless be stated with appropriate caution. A systematic review of 25 randomized trials of exercise-based prehabilitation across cancer populations found no clear effect between groups and graded the overall certainty of evidence as very low, citing study limitations and imprecision (20). Trials have largely been small and heterogeneous in their protocols, and octogenarians in particular have rarely been enrolled in sufficient numbers to support age-specific conclusions. Prehabilitation is therefore best regarded at present as a biologically plausible and guideline-endorsed strategy (21) whose effect size in the oldest surgical patients remains undefined, and it should not be presented to a frail octogenarian as a reliable route to operability. Structured perioperative pathways extend the same logic through the operation and beyond, since enhanced recovery guidance for lung surgery bundles preoperative counseling, nutritional screening, smoking cessation, prehabilitation for high-risk patients, opioid-sparing analgesia, minimally invasive surgery, early drain removal, and early mobilization into a coherent program (22). For a patient with marginal reserve, the difference between an operation that is tolerated and one that is not may lie less in the resection itself than in the pathway surrounding it.

The multidisciplinary team is the natural home for this process, and its composition matters as much as its existence. Guidance on fitness for radical treatment already places these patients within specialized multidisciplinary settings (9), but in the oldest cohorts the team benefits from geriatric input alongside surgical, oncologic, and radiation expertise, since the deficits that determine tolerance are often outside the thoracic surgeon's usual field of view (8, 17). Embedding assessment in a standing pathway rather than leaving it to individual discretion also makes the resulting decisions auditable, which is a precondition for improving them (22).

Selection must finally incorporate what the patient wants. Seriously ill older adults frequently decline treatments that carry a meaningful risk of functional or cognitive impairment, even when those treatments could extend life (23). A resection that is technically successful but leaves a patient dependent, against their stated priorities, is not a good outcome. Eliciting goals explicitly is therefore part of selection, not an afterthought to it, and the elicitation should be documented with the same rigor as a physiologic measurement. In practice this conversation has a structure that can be taught. A three-step approach of introducing that a genuine choice exists, describing the options and their trade-offs, and then helping the patient deliberate on what matters most to them translates the principle into a reproducible consultation (24). Several features matter particularly at this age. Outcomes should be framed in absolute terms over a horizon the patient recognizes, and expressed as function, independence, and time at home rather than as survival percentages alone. Competing risk should be named openly, since a patient who understands that another condition may determine their life expectancy is better placed to weigh an operation. A caregiver should be present where the patient wishes, as caregivers shape both the decision and the recovery. Sensory impairment, health literacy, and the cognitive load of a single long consultation argue for written summaries and, where possible, a second visit before a decision is finalized. Structuring the discussion around a geriatric assessment supports this directly: in a cluster-randomized trial, providing oncologists with a geriatric assessment summary increased the number of aging-related concerns discussed and improved patient and caregiver satisfaction with communication (25). Where the patient's priorities and the technically feasible operation diverge, the priorities should govern, and the reason should be recorded.

## Discussion

For octogenarians, survival is a necessary but insufficient measure of a good result. Functional independence, preserved cognition, and time spent at home rather than in hospital weigh at least as heavily. Postoperative delirium illustrates the point: it is common, serious, and frequently under-recognized in older hospitalized patients, it signals reduced cerebral reserve, and it is associated with lasting cognitive decline, yet it is invisible to conventional surgical endpoints such as operative mortality (26). Patient-centered measures such as days alive and out of hospital capture precisely what survival curves omit, integrating length of stay, readmission, and early death into a single validated count (27). A selection strategy built on reserve and priorities should be paired with outcome measures that reflect those same priorities.

Several gaps follow from this argument and define a research agenda. Randomized trials of resection continue to under-enroll the oldest patients, so the evidence guiding decisions in the ninth decade remains largely observational and vulnerable to the very selection effects it seeks to describe. Frailty instruments have been validated for prognosis but not prospectively tested as the basis for allocating treatment intensity in thoracic surgery. Reporting standards could help immediately: series in older patients should describe not only the patients who were operated on but also those who were assessed and not offered an operation, together with the instrument and threshold used, so that careful selection becomes legible and comparable across centers.

The recurring question of whether a patient is too old to operate is, in the end, the wrong one. The better question is what intensity of treatment matches this particular patient's physiologic reserve and personal goals, how that judgment can be made reproducible through structured assessment and shared decision-making, and how success should be defined once the operation is done. The supporting evidence, the graded treatment options, and the assessment tools all already exist. What remains is the discipline to treat age as a starting fact rather than a deciding one, so that careful selection becomes something we can describe, teach, and audit rather than simply assert.

## Data Availability

The original contributions presented in the study are included in the article; further inquiries can be directed to the corresponding author.
